# Risk factors for, metrics of, and consequences of access to veterinary care for companion animals: A scoping review

**DOI:** 10.1371/journal.pone.0325455

**Published:** 2025-05-30

**Authors:** Annette O’Connor, Sarah Ceridwen Totton, Michelle Hernandez, Emily Meyers, Kelley Meyers, Hilda Mejia Abreu, Nathaniel Spofford, JoAnn Morrison

**Affiliations:** 1 Department of Large Animal Clinical Sciences, College of Veterinary Medicine, Michigan State University, East Lansing, Michigan, United States of America; 2 Private Consultant, Guelph, Ontario, Canada; 3 Michigan State University Veterinary Medical Center, Michigan State University, East Lansing, Michigan, United States of America; 4 Mars Veterinary Health, Vancouver, Washington, United States of Amierica; Quinnipiac University, UNITED STATES OF AMERICA

## Abstract

**Background:**

Barriers to accessing veterinary care can be challenging for companion-animal caregivers and may lead to preventable health conditions or even death of pets.

**Objectives:**

We conducted a scoping review to: 1) catalog the definitions of access to veterinary care (A2VC) used by researchers, 2) identify risk factors for and consequences of A2VC, and 3) map the risk factors onto dimensions of access to care (affordability, availability, accessibility, accommodation, acceptability).

**Eligibility criteria:**

Primary research on companion animals not involved in commercial enterprises (e.g., horse racing) examining consequences of and/or risk factors for A2VC for which the full text was available in English.

**Sources of evidence:**

PubMed (1996–6 July 2023) and CAB Abstracts (1973–13 July 2023, Web of Science^TM^) were searched. Additionally, a topic expert (KM) identified relevant references. Two reviewers independently screened titles/abstracts and full texts of potentially relevant references. Forward and backward citation searches were also conducted on all eligible studies using Citation Chaser.

**Charting methods:**

Risk factors were categorized and mapped to the five dimensions of access to care. An evidence gap map was created using the risk factor studies.

**Results:**

Fifty-one references describing fifty-two relevant studies were included. Forty-one studied risk factors associated with A2VC, and twelve studied consequences of A2VC. (One study examined both risk factors and consequences.) The majority of risk factors examined were demographic. The majority of outcomes measured were pet-centric. No relevant studies focused on pet horses, representing a gap in the literature.

**Conclusions:**

Consensus needs to be reached on how A2VC is defined and measured to help reduce research wastage and strengthen impact of future studies on improving A2VC. Future studies of risk factors for A2VC should focus on creating a risk-mapping framework specific for A2VC, distinguishing factors that are susceptible to change and those which are not.

## 1. Introduction

### 1.1. Rationale

Companion animals, particularly cats and dogs, play an essential role in the lives of many people, providing love, companionship, and a sense of purpose [1–3]. Studies have shown that pets can improve their caregivers’ mental health by reducing stress and anxiety, increasing social interaction and physical activity, and decreasing feelings of loneliness. However, accessing veterinary care can be a significant challenge for many caregivers, and this may have a negative impact on both the welfare of the animals and the physical and mental health of their caregivers [4]. The problem is that access to care is more complex than physical proximity to a care provider. Access to care in the human health field has been grouped into five dimensions: affordability, availability, accessibility, accommodation, and acceptability (Table 1) [5,6].

**Table 1:** Dimensions of access to veterinary care for companion animals (based on dimensions of access to human health care [5,6]).

Dimension	What is it?
Affordability	Cost for services, caregiver’s knowledge of cost, ability and willingness to pay, caregiver’s perception of worth
Availability	Ratio and type (e.g., emergency, specialty) of veterinary services to companion animals and caregiver needs in a defined geographic area
Accessibility	Distance between the caregiver and service(s), ease/availability/cost of transport of animal to service or veterinarian to caregiver
Accommodation	How services are set up to meet caregiver preferences/constraints (i.e., office hours, walk-in appointments, wait times for appointments, ability/ease of getting in touch with veterinarian)
Acceptability	Caregiver’s attitudes toward veterinary clinic, staff and services, veterinary clinic staff’s attitudes toward caregiver

Access to veterinary care (A2VC) is an emerging field, and research in this area has the potential to shed light on the root causes of barriers to adequate veterinary care and to inform strategies to address them [4]. Such research aligns with broader movements within the veterinary profession to promote equity and inclusivity in the membership, leadership, and organization [7]. Much of the current A2VC research has focused on identifying the extent and nature of the problem, including geographic areas and populations most affected by limited A2VC [8–13]. However, to make meaningful progress toward addressing this issue, research must move beyond identifying the problem, to assessing interventions that can improve A2VC in underserved areas.

The absence of clear metrics for measuring the impact of interventions to improve A2VC represents a significant challenge. Identifying outcomes that are both relevant and measurable and that are considered important to researchers and the involved community is critical for evaluating the effectiveness of interventions. One issue we have observed is that A2VC is defined in many ways in veterinary sciences. Inconsistencies in defining the problem can create issues, such as lack of consensus about the problem being solved or an inability to compare the relative impact of different interventions. In addition, variability in how outcomes are defined and measured can make it difficult or impossible to synthesize and apply the results of different studies of animal welfare and health [14,15]. It is our belief that companion animals, their caregivers, and the veterinary services industry will all benefit from a better understanding of the current knowledge of A2VC.

Scoping reviews are an evidence synthesis tool that can be used to clarify definitions of concepts within a particular topic area and to identify gaps in the current body of research [16]. At the time we conducted our literature search, screening and data charting, there were no published scoping reviews on the topic of risk factors and consequences of A2VC. Subsequently, in October of 2023, a scoping review of A2VC was published [17]. The findings of this review in the context of our own review, are covered in the Discussion section of the current study.

### 1.2. Objectives

The objectives of our scoping review were to: 1) catalog the definitions of the variable “access to veterinary care in companion animals” used by researchers, 2) identify the metrics used as risk factors for A2VC and consequences of A2VC at the individual- and group- (ecological) level, and 3) map the risk factors onto five dimensions of A2VC (affordability, availability, accessibility, accommodation, and acceptability).

## 2. Materials and methods

This scoping review is reported using the PRISMA Extension for Scoping Reviews [18].

### 2.1. Registration

The protocol for the current study is available online at Systematic Reviews for Animals and Food (SYREAF) at https://syreaf.org/wp-content/uploads/2023/07/Final-Acess-to-veterinary-care-A2VC-scoping-review-protocol-1.pdf.

### 2.2. Eligibility criteria

Eligible population: Companion animals, which may consist of dogs, cats, pocket pets, equids, or any other companion animal, including free-roaming, unowned, or stray. We did not translate studies; therefore, only studies for which the full text was available in English were eligible for inclusion. Animals that were involved in commercial enterprises (e.g., race horses, herding dogs) were not eligible. Production (food supply) animals were likewise not eligible.

Eligible concept: In order to be eligible the “veterinary care” in question had to be therapeutic or preventive (e.g., vaccines) and administered by a professional (i.e., not by the caregiver; e.g., If a caregiver bought a flea collar at a pet store and put it on their pet, this would not qualify as “veterinary care”). Studies of cosmetic grooming or nutrition (pet food, pet food banks, etc.) were not eligible.

Eligible study designs/ context: Studies investigating the consequences of A2VC (i.e., A2VC as an explanatory variable) were eligible. Studies investigating risk factors for A2VC were also eligible. Therefore eligible study designs comprised observational studies (cross-sectional, cohort, case-control), randomized controlled trials, and quasi-experiments (before-and-after trials). Ecological studies were also eligible. Manuscripts structured solely as reviews were not eligible. Studies for which the unit of interest was veterinary clinic staff were not eligible.

### 2.3. Information sources

A literature search was conducted in PubMed on 6 July 2023 (from 1996 to date of search) and in CAB Abstracts on 13 July 2023 (from 1973 to date of search) in the Michigan State University Web of Science^TM^ interface. We searched only these two databases because, based on the authors’ extensive experience conducting systematic and scoping reviews in veterinary medical topic areas, these were considered sufficient to cover the relevant literature. We also performed forward and backward citation searches on all references that passed full-text screening using a Citation Chaser tool (https://estech.shinyapps.io/citationchaser/).

We had planned, as per our protocol, to search the following sources: ASPCA and University of Minnesota Access to Veterinary Care Conference held from 17 to 19 October 2022, ASPCA Maddie’s® Shelter Medicine Conference (https://www.vet.cornell.edu/aspca-cornell-maddies-shelter-medicine-conference), Animal Care Expo (https://humanepro.org/expo), and Best Friends National Conference, and www.humananimalsupportservices.org/. However, none of these events had publicly available proceedings to search.

Additional potentially relevant references were identified by our topic expert (KM) based on her expertise in the field.

### 2.4. Search

The search included 1) the (companion animal) population and 2) the A2VC concept (which included some caregiver aspects). The search in PubMed is shown in Table 2. The corresponding search performed in CAB Abstracts is shown in S1 Table. No limits were applied to the searches.

**Table 2:** Search strings used in a PubMed search conducted on 6 July 2023 for a scoping review of risk factors for, metrics of, and consequences of access to veterinary care for companion animals.

String	Search	No. hits
1	“access to veterinary care”[Title/Abstract] OR “access to care”[Title/Abstract] OR “low income”[Title/Abstract] OR “underserved”[Title/Abstract] OR “Unserved”[Title/Abstract] OR “socioeconomic”[Title/Abstract]	231,405
2	((“animals”[MeSH Terms:noexp] OR “animal”[All Fields] OR (“horse s”[All Fields] OR “horses”[MeSH Terms] OR “horses”[All Fields] OR “horse”[All Fields]) OR (“rabbit s”[All Fields] OR “rabbits”[MeSH Terms] OR “rabbits”[All Fields] OR “rabbit”[All Fields]) OR (“ferrets”[MeSH Terms] OR “ferrets”[All Fields] OR “ferret”[All Fields]) OR “guinea pig”[All Fields] OR (“veterinary”[MeSH Subheading] OR “veterinary”[All Fields]) OR “cat”[All Fields] OR (“dogs”[MeSH Terms] OR “dogs”[All Fields] OR “dog”[All Fields]) OR (“pet”[All Fields] AND “Or”[All Fields])) AND “companion animal”[All Fields]) OR (“canine s”[All Fields] OR “dogs”[MeSH Terms] OR “dogs”[All Fields] OR “canine”[All Fields] OR “canines”[All Fields]) OR (“cats”[MeSH Terms] OR “cats”[All Fields] OR “felines”[All Fields] OR “felidae”[MeSH Terms] OR “felidae”[All Fields] OR “feline”[All Fields]) OR “pony”[All Fields] OR (“donkey s”[All Fields] OR “equidae”[MeSH Terms] OR “equidae”[All Fields] OR “donkey”[All Fields] OR “donkeys”[All Fields]) OR (“ponies”[All Fields] AND “Or”[All Fields] AND (“equidae”[MeSH Terms] OR “equidae”[All Fields] OR “mule”[All Fields]))	587,448
3	#1 AND #2	708

### 2.5. Selection of sources of evidence

The search results were downloaded in a tagged format into bibliographic software as RIS files. Separate files were obtained for the PubMed and CABI searches. These files were imported into online systematic review software (DistillerSR®) (Evidence Partners, Ottawa, ON, Canada) and de-duplicated using DistillerSR’s duplicate detection tool, followed by a manual duplication check in Excel. Before screening began, the reviewers assigned to each phase of the review underwent training to ensure consistent screening and data collection, using forms created in DistillerSR®.

In the first round of screening, the titles and/or abstracts of each citation were assessed for eligibility by two reviewers working independently using the following screening question:

1. Does the study describe primary research that either assesses risk factors for, or consequences of, any metric of access to veterinary care in companion animals?

- Yes (include for full-text evaluation)
- Unclear (include for full-text evaluation)
- No (exclude with no further review)
- No, but this looks like a potentially relevant review (exclude with no further review)

A pre-test of the title/abstract screening form was conducted by all reviewers on the first 185 citations to ensure clarity and consistency of understanding of the screening question. Citations were excluded if both reviewers responded “no” to the screening question. If both reviewers said “yes” (or “unclear”), the citation advanced to full-text assessment. All conflicts were resolved prior to exclusion via discussion between the two reviewers, or, if consensus could not be reached, by consulting a third reviewer (SCT or AOC).

The full text of all citations that passed title/abstract screening were procured from the Michigan State University Library or online sources, if the PDF was publicly accessible. The full-text screening form was pre-tested on one reference by all reviewers. Subsequently, two reviewers independently evaluated each of the full-text articles for eligibility with any disagreements resolved by consensus. When a consensus could not be reached, a third reviewer was consulted. The questions in the full-text eligibility assessment form were as follows:

1. Is the full text available?
  - Yes (proceed to Q2)
  - No (exclude with no further review)
2. Is the full text in English?
  - Yes (proceed to Q3)
  - No (specify language) (exclude with no further review)
3. Is the study about access to veterinary care for companion animals (including unowned/stray/free-roaming)?
  - Yes (proceed to Q4)
  - No (exclude with no further review)
4. At what level does the access to veterinary care metric apply?
  - Individual (proceed to Q5)
  - Household (e.g., distance to a veterinary clinic) (proceed to Q5)
  - Postal Code (e.g., number of veterinary clinics in the postal code) (proceed to Q5)
  - City/County/State/Province (e.g., number of veterinary practices in the state) (proceed to Q5)
  - Other (e.g., veterinary clinic employees) (specify) (exclude with no further review)
5. Correct design. Is the study design of interest?
  - The study compares risk factors that impact A2VC (proceed to data charting)
  - The study compares the consequences of A2VC (proceed to data charting)
  - The study is a relevant review article (exclude with no further review)
  - None of the above (e.g., focus group, no comparison group, non-relevant review) (exclude with no further review)

### 2.6. Data charting process

The data charting form was drafted in DistillerSR® and pre-tested with all reviewers on three references. Subsequently, each reference that passed full-text screening was categorized by two reviewers working independently, with any conflicts resolved by discussion or, if consensus could not be reached, by consulting a third reviewer. Investigators of included studies were not contacted to obtain or confirm data.

### 2.7. Data items

A list of the variables for which data were collected is provided in Table 3.

**Table 3:** Data charting form for a scoping review of risk factors for, metrics of, and consequences of access to veterinary care for companion animals.

Question	Type	Answer options
**Q1**. What is the Study ID? (If the reference describes only one study, the Study ID is the same as the Ref ID.)	Text	
**Q2.** What type of study population is this?	Radio	Individual/Household County/Parish/State/Province, etc.
**Q3.** What was the authors’ definition of the population studied?	Text	Quote the relevant text from the reference
**Q4.** In which country/countries was the study conducted?	Checkbox	A list of countries
**Q5.** In what year(s) was the study conducted?	Text	
**Q6.** What was the authors’ definition of access to veterinary care?	Text	Quoted text from the reference
**Q7.** Is this a study of risk factors affecting A2VC (e.g., demographics, socioeconomic status, etc.) or is this a study of the consequences of A2VC?	Checkbox	Risk factors Consequences
**Q8.** If this was a study of risk factors affecting A2VC, list all of the risk factors assessed.	Checkbox	A list of risk factors
**Q9.** If this was a study of consequences of A2VC, list those consequences.	Checkbox	A list of consequences
**Q10.** What risk factor was studied?	Text	
**Q11.** What type of risk factor was this?	Checkbox	Human Animal
**Q12.** Was the risk factor self-reported?	Radio	Yes No
**Q13.** What dimension(s) of access to veterinary care does the risk factor map to? (Select all that apply.)	Checkbox	Affordability Availability Accommodation Accessibility Acceptability
**Q10**, **Q11**, **Q12**, and **Q13** were repeated for each risk factor studied.		

### 2.8. Critical appraisal of individual sources of evidence

As this was a scoping review, we did not critically appraise the included studies.

### 2.9. Synthesis of results

After extensive discussion amongst the authors of the current study, each risk factor was categorized and mapped to one or more of the five dimensions of access to care (affordability, availability, accessibility, accommodation, and acceptability). Data from the risk factor studies was coded in EPPI-Reviewer version 4 [19] and subsequently exported to EPPI-Mapper [20] to create an Evidence Gap Map.

## 3. Results

### 3.1. Selection of sources of evidence

**Fig 1:**
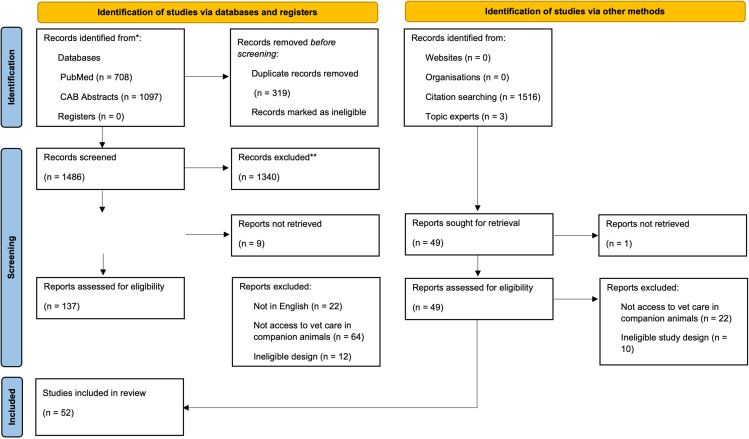
PRISMA flow diagram [21] of the number of records identified, screened, and included in a scoping review of risk factors for, metrics of, and consequences of access to veterinary care for companion animals.

The number of records identified from each source, screened, assessed for eligibility, excluded (with reasons), and included in the review are shown in Fig 1. Of the 22 references that were excluded at the full-text stage because they were not in English, 15 were in Portuguese, 4 were in Spanish, 1 was in French, 1 was in Turkish, and 1 was in Dutch. A list of citation information for all records excluded at the full-text stage, along with reasons for exclusion, are provided in S2 Table.

### 3.2. Characteristics of sources of evidence

Data from 51 references describing 52 studies were charted. Complete citation information for all included studies is provided in S3 Table. Of the 52 included studies, 43 studies were at the individual/household level while 9 studies were at a higher level (postal code (3 studies), zip code (1 study), neighborhood (1 study), census tract (1 study), district (1 study), county (1 study), and village (1 study)). The studies were conducted in the USA (19 studies), Brazil (5 studies), Australia (3 studies), United Kingdom (4 studies), Canada (2 studies), Haiti (2 studies), Tanzania (2 studies), and 1 study each was conducted in Burkina Faso, Cameroon, Chile, China, Democratic Republic of Congo, Ghana, Japan, Malawi, Mexico, Nigeria, Peru, Philippines, Republic of Ireland, Uganda, Vietnam, and Zambia. One study did not report the country in which it was conducted. (Note: Some studies were conducted in more than one country.)

Table 4 reports the year(s) in which each included study was conducted. Thirteen studies did not report the year in which the study was conducted.

**Table 4:** Years of conduct of studies included in a scoping review of risk factors for, metrics of, and consequences of access to veterinary care for companion animals.

Year(s) of conduct	Number of studies
1992	1
2003	1
2009	1
2010	2
2011	1
2013	1
2005–2014	1
2014	1
2011–2015	1
2014–2015	2
2011–2016	1
2016	4
2008–2017	1
2017	2
2008–2018	1
2016–2018	1
2017–2018	2
2018	2
2018–2019	2
2014–2020	1
2020	7
2019–2021	1
2020–2021	1
2021	1
2022	1

Forty-one studies were of risk factors associated with access to veterinary care, while twelve studies were of consequences of access (or lack of access) to veterinary care. One study examined both risk factors and consequences of access to care.

### 3.3. Critical appraisal within sources of evidence

As this was a scoping review, critical appraisal within sources of evidence was not done.

### 3.4. Results of individual sources of evidence

Authors’ definitions of the study population and access to veterinary care for each included study are reported in S3 Table.

### 3.5. Consequences of access to veterinary care

The consequences of (lack of) access to veterinary care examined in the included studies comprised: Dog rabies vaccination coverage (three studies), canine visceral leishmaniasis infection (two studies), prevalence of spay/neuter (two studies), and one study each examined age at desexing in cats, average age in dogs, body condition in dogs, prevalence of overweight and obesity in dogs, canine health-related quality of life, clusters of canine parvovirus, pet caregiver’s trust in the veterinary profession, prevalence of desexing in cats, prevalence of heartworm prevention, prevalence of flea/tick prevention, deworming status in dogs, prevalence of injury/illness care within the past year, prevalence of preventive care within the past year, prevalence of up-to-date vaccinations, prevalence of vaccinations other than rabies, prevalence of problem behaviors in dogs, prevalence of obedience training, relinquishment of pet to a shelter, and use of an animal poison control center tele-triage service.

### 3.6. Risk factors for access to veterinary care

Tables 5–7 report the pet-related, caregiver-related, and household-/other-related risk factors, respectively, examined in the included studies, along with their corresponding mapping to the five dimensions of access to care for companion animals.

**Table 5:** Pet-related risk factors mapped to the dimensions of access to veterinary care for companion animals.

Risk factor (no. of studies)	Affordability^a^	Availability^b^	Accessibility^c^	Accommodation^d^	Acceptability^e^
Species of pet (3)	X		X	X	
Age of pet (13)	X		X		
Breed of pet (6)	X				
Size of dog (1)	X		X		
Sex of pet (10)	X				
Spay/neuter status of pet (6)	X				
Pregnancy/lactating status of dog (2)	X				X
Primary purpose of pet (e.g., companion, guard) (3)	X				
Source of pet (gift, purchased, found, adopted, progeny of existing pet, etc.) (8)	X				
Pet is owned vs stray/dog resides in multiple households/confinement status (of pet) (9)	X		X		
Dog is registered (licensed) (1)	X				
Dog is accustomed to leash (2)	X		X		X
Location where the dog sleeps at night (1)	X				
Pet has a wellness plan (1)	X				
Pet’s vaccination history (1)	X				
Whether the dog has had a major illness in the past (1)	X				
Number of people bitten by pet (dog) (1)					X
Dog health (diseased vs healthy) (2)	X	X	X		

^a^Affordability comprises cost for services, caregiver’s knowledge of cost, ability and willingness to pay, and caregiver’s perception of worth.

^b^Availability comprises ratio and type (e.g., emergency, specialty) of veterinary services to companion animals and caregiver needs in a defined geographic area.

^c^Accessibility comprises distance between caregiver and service(s), ease/availability/cost of transport of animal to service or veterinarian to caregiver.

^d^Accommodation comprises how services are set up to meet caregiver preferences/constraints: Office hours, walk-in appointments, wait times for appointments, and ability/ease of getting in touch with veterinarian.

^e^Acceptability comprises caregiver’s attitudes toward veterinary clinic, staff and services, veterinary clinic staff’s attitudes toward caregiver.

**Table 6:** Caregiver risk factors mapped to the dimensions of access to veterinary care for companion animals.

Risk factor (no. of studies)	Affordability^a^	Availability^b^	Accessibility^c^	Accommodation^d^	Acceptability^e^
Age (15)	X		X		
Gender (13)	X				
Race (5)					X
Ethnicity (5)					X
Race/ethnicity combined (1)					X
Caregiver is a member of a vulnerable population (1)					X
Social Vulnerability Index (incorporates socioeconomic status, household composition and disability, minority status and language and housing type and transportation) (1)	X	X	X	X	X
Socioeconomic status/income (21)	X				X
Caregiver qualifies for housing benefit/SNAP/WIC, etc. (2)	X				X
Caregiver receives public assistance (1)	X				X
Financial fragility (whether an unexpected $1000 veterinary bill would cause financial stress) (2)	X				
Caregiver has pet insurance (2)	X				
Employment status (employed, unemployed, student) (5)	X				
Occupation (1)	X		X		
Caregiver employed in human-/animal-health-related field (1)	X				
Caregiver also owns livestock (1)	X	X	X		
Marital status of caregiver (2)	X				
Religion (2)					
Education (14)	X		X		
Languages spoken (1)				X	
Who is the caregiver (head of household/other) (2)	X		X		
Self-efficacy of caregiver (confidence that one’s actions will lead to the desired outcome) (1)	X				X
Caregiver’s attitude (1)	X				X
Dog’s caregiver is able to interact with other dog caregivers (1)	X				
Having a veterinary clinic your family goes to (1)			X		
Caregiver’s vaccination history (1)	X	X			X
Lack of trust in veterinarians (1)	X				
Caregiver provides healthcare to animal themselves (1)	X				
Attitude toward pet (incl. attachment) (4)	X				
Caregiver’s risk perception regarding future dog illness (1)	X				
Expenditure on food, toys and other non-healthcare-related items (1)	X				
Knowledge/awareness of rabies (4)	X				
Rabies status of last place lived in before moving to the study area (1)	X				
Presence of someone in the household who has known someone with rabies (1)	X				
Knowledge about mandatory dog rabies vaccination (2)	X				
Perception of cost of rabies vaccination (1)	X				
Length of time caregiver has lived in the area (1)	X				
Knowledge of veterinary clinic location (1)			X		
Thinks veterinary care is too expensive (2)	X				
Unable to find a veterinarian nearby (1)		X	X		
Unable to transport animal to veterinarian (4)			X	X	
Unable to understand veterinarian (1)				X	
No time to bring animal to veterinarian (1)			X	X	
Caregiver thinks veterinary clinic isn’t open at convenient hours (2)				X	

^a^Affordability comprises cost for services, caregiver’s knowledge of cost, ability and willingness to pay, and caregiver’s perception of worth.

^b^Availability comprises ratio and type (e.g., emergency, specialty) of veterinary services to companion animals and caregiver needs in a defined geographic area.

^c^Accessibility comprises distance between caregiver and service(s), ease/availability/cost of transport of animal to service or veterinarian to caregiver.

^d^Accommodation comprises how services are set up to meet caregiver preferences/constraints: Office hours, walk-in appointments, wait times for appointments, and ability/ease of getting in touch with veterinarian.

^e^Acceptability comprises caregiver’s attitudes toward veterinary clinic, staff and services, veterinary clinic staff’s attitudes toward caregiver.

**Table 7:** Housing and other risk factors mapped to the dimensions of access to veterinary care for companion animals.

Risk factor (no. of studies)	Affordability^a^	Availability^b^	Accessibility^c^	Accommodation^d^	Acceptability^e^
*Housing Factors*					
Number of pets in the household (7)	X		X		
Housing insecurity (1)	X		X		X
Number of people in household (9)	X				
Presence of children (< 15 years old) in the household (3)	X		X		
Number of children (< 5 years old) in the household (1)	X		X		
Type of home (apartment, house, etc.) (5)	X	X			
Home has internet access (1)	X		X	X	
Sewage system for home (1)	X				
Public water supply in home (1)	X				
Public garbage collection service for home (1)	X				
Human population density in area of residence (1)		X	X		
Urban vs rural residence (4)		X	X		
Housing density in the area of residence (1)		X	X		
Geographic location (12)	X	X	X		
Distance from household to nearest static dog vaccination point (4)			X		
Land cover (forest/grassland/cropland/settlement) (1)		X	X		
Land use (farm/forest/industrial/nature reserve/residential/scrub) (1)		X	X		
*Other factors*					
COVID-19 restrictions/lockdown (2)	X		X	X	
COVID-19 pandemic (3)	X		X	X	
Pets for Life (Humane Society of the United States’ Pets for Life program (addresses the issue of access to pet support services by offering no cost or heavily subsidized pet care services, providing transportation to and from appointments, employing bilingual staff members, building relationships with pet caregivers in the community, and partnering with local companion animal service organizations to provide services) (1)	X		X	X	X
Service provider’s cultural competence (1)				X	
Mass dog vaccination opportunity (1)			X		

^a^Affordability comprises cost for services, caregiver’s knowledge of cost, ability and willingness to pay, and caregiver’s perception of worth.

^b^Availability comprises ratio and type (e.g., emergency, specialty) of veterinary services to companion animals and caregiver needs in a defined geographic area.

^c^Accessibility comprises distance between caregiver and service(s), ease/availability/cost of transport of animal to service or veterinarian to caregiver.

^d^Accommodation comprises how services are set up to meet caregiver preferences/constraints: Office hours, walk-in appointments, wait times for appointments, and ability/ease of getting in touch with veterinarian.

^e^Acceptability comprises caregiver’s attitudes toward veterinary clinic, staff and services, veterinary clinic staff’s attitudes toward caregiver.

### 3.7. Synthesis of results

An interactive evidence gap map, grouping the risk factors (pet factors, caregiver factors, household factors, and other factors) and outcomes (pet-related outcomes, caregiver-related outcomes, housing-related outcomes, and clinic-centric outcomes) is shown in S1 Fig.

## 4. Discussion

### 4.1. Summary of the evidence

#### 4.1.1. Definitions and metrics of access to veterinary care.

Although we did not find a single, universally applied metric of access to care in the relevant studies in our review, the most commonly used metric (16 studies) was rabies vaccination coverage. While this may be relevant to certain geographic locations (i.e., where dog rabies is endemic), it is less germane in other locations (e.g., USA). Other metrics, such as spay/neuter rate, have a cultural and subjective aspect that cannot always be ascribed to lack of access to care.

In the primary research studies included in our review, we found no recognized or commonly used definitions of access to veterinary care. However, a recent scoping review [17], published subsequent to our literature search, defined “underserved” with respect to lack of access to adequate veterinary care (in particular for dogs) applied to areas, communities, and animals. According to Roberts and others [17], “an Animal Health Underserved Area is a community or region that lacks adequate, accessible, and safe animal health services such that the community self-identifies that dog health and welfare and human/ community health are negatively impacted,” “an Animal Health Underserved Individual is a person who has inadequate access to animal health services such that they identify that their dog’s health and welfare or their own personal health and well-being are negatively impacted,” and “an Animal Health Underserved Population is a group or population of dogs that has inadequate access to animal health services such that, collectively or individually, the dogs’ health and welfare are negatively impacted.” We believe that, going forward, primary research studies on access to veterinary care in companion animals should utilize these definitions, perhaps expanding on them to include other species of companion animals and to consider application to a wider geographic area (the Roberts et al. [17] review covered the USA, Canada, and Australia only) to help achieve uniformity across studies and to help, as Roberts and others [17] suggest, develop a scoring system to assess severity of need to enable comparability of degree of access to care between and within studies.

#### 4.1.2. Mapping risk factors to the dimensions of access to care.

We opted to use a previously published dimensional framework described by Penchansky and Thomas [5], for mapping access to care. However, because this framework was created in the context of human health care access, applying it to veterinary health care access presented a challenge in certain ways. For instance, the affordability dimension encompasses both willingness and ability to pay. Ability to pay is an important barrier to veterinary care. Willingness to pay is also an issue in veterinary care, particularly for preventive care, which people may not see as valuable [22,23]. Ability and willingness to pay are two separate problems with separate solutions and by mapping them onto the single dimension of affordability, some nuance may be lost.

Mapping the risk factors to the five dimensions of access to care was a complex task involving many subjective judgments and much discussion amongst the authors of the current study. Of the 18 pet-related risk factors, 9 (50%) were difficult to map (number pets in the household, species, breed, size, purpose, pregnancy/lactating status, accustomed to leash, and pet health), which is not surprising, given that the mapping framework was designed for mapping human health care access. In contrast, a lower percentage of the caregiver-related factors were challenging to map (13/44; 30%); these factors included the demographic factors (age, race/ethnicity, religion, gender, self-efficacy, and occupation), economic factors (socioeconomic status, pet insurance) and factors such as whether the caregiver owns livestock, interacts with other dog caregivers, vaccination history, rabies status of previous place lived in and whether they were able to transport their pet to the veterinarian. The translation of human demographic factors from a healthcare access standpoint to a veterinary care access standpoint is a complex one, and, rather than debate where each factor should be mapped to the current framework, it might be more pertinent to consider which factors are more salient in a different framework designed specifically for veterinary care access. Of the household-related risk factors, 4 out of 17 (25%) were challenging to map (internet access, sewage system present, housing density in area of residence, and geographic location).

#### 4.1.3. Overall findings.

Although we did not exclude equine companion animals from our review, we did not find any studies focusing on access to care for companion horses, and this represents a gap in the primary literature. Additionally, though less surprisingly, we likewise did not find any studies focusing on pocket pets.

Our study identified the need for a risk-mapping framework specific to access to veterinary care. In particular, one that distinguishes between the caregivers’ ability and willingness to pay. Ideally, this framework would also distinguish between which factors are susceptible to intervention (e.g., economic risk factors and the attitudes, perceptions, and knowledge of caregivers) and those which are not (e.g., pet and caregiver demographic factors). It might also be useful if the framework also maps factors by which sector has control over them (e.g., veterinary-controlled vs caregiver-controlled).

#### 4.1.4. Evidence gap map.

The majority of outcomes measured in the included studies were pet-centric, possibly because these outcomes are easier to measure than clinic-centric or caregiver-centric outcomes, and there are fewer issues over confidentiality. Demographic risk factors (for pets and caregivers and location of household) were commonly assessed, possibly because these factors are easy to measure. However, the issue with demographic factors is that they tend to be immutable, or at least, difficult to change. Economic risk factors and the attitudes, perceptions, and knowledge of caregivers are probably the most useful factors to study as they can be susceptible to improvement with various interventions.

### 4.2. Limitations

Because our review was restricted to references for which the full text was available in English, 22 potentially relevant references were excluded at the full-text stage because they were not in English. As a consequence, our results are biased towards countries in which authors tend to publish studies in English.

Another limitation of our review is that some of our risk factor mapping was subjective (as outlined above) and open to debate.. Although we used a previously published framework for risk factor mapping, this framework was designed for human health studies and thus presented challenges in applying it to veterinary care access. There appears to be a need for a more suitable access framework, in particular to address the risk factors that were particularly difficult to map using the human health framework (race, ethnicity, religion, etc.). This work was designed to highlight the current state of knowledge, to identify gaps in the literature, and to initiate discussion on alignment and lexicon, to encourage and stimulate ongoing and robust research in this area.

## 5. Conclusions

Although it is probably unrealistic to create a definition of access to veterinary care that would be applicable worldwide, getting alignment on a commonly accepted definition, perhaps specific to certain parts of the world, would be major progress in and of itself.

This review identified a need for a risk factor mapping framework specific to access to veterinary care, specifically, one which distinguishes between which factors are susceptible to intervention (e.g., attitudes, perceptions, and knowledge of caregivers) and those which are not (e.g., pet and caregiver demographic factors).

We plan to use the information from this review to conduct a consultative project with experts and community members to capture opinions about the dimensions of A2VC captured by the metrics and value (high value/ low value), mutable and immutable consequences, and individual- and group-level metrics of impact. We will then develop a proposal for a core set of outcomes and definitions for A2VC and dimensions for use by future researchers to design studies that can assess the impact of interventions on veterinary care access.

## Supporting information

S1 Table Search strings used in a CAB Abstracts search conducted on 13 July 2023 for a scoping review of risk factors for, metrics of, and consequences of access to veterinary care for companion animals. (DOCX)

S2 Table Records excluded after full-text evaluation, with reasons for exclusion, in a scoping review of risk factors for, metrics of, and consequences of access to veterinary care for companion animals. (XLSX)

S3 Table Complete citation information and authors’ definitions of the study population and definitions of access to veterinary care for all included studies in a scoping review of risk factors for, metrics of, and consequences of access to veterinary care for companion animals. (XLSX)

S1 Fig An interactive evidence gap map, grouping the risk factors (pet factors, caregiver factors, household factors, and other factors) and outcomes (pet-related outcomes, caregiver-related outcomes, housing-related outcomes, and clinic-centric outcomes) from a scoping review of risk factors for, metrics of, and consequences of access to veterinary care for companion animals. (HTML)

S1 File PRISMA Scoping Review Checklist for revised manuscript. (PDF)
