# Correction: Risk factors for, metrics of, and consequences of access to veterinary care for companion animals: A scoping review

**DOI:** 10.1371/journal.pone.0333116

**Published:** 2025-09-23

**Authors:** The PLOS One Staff

Fig 1 contains several errors. Please see the correct Fig 1 here.

**Fig 1:**
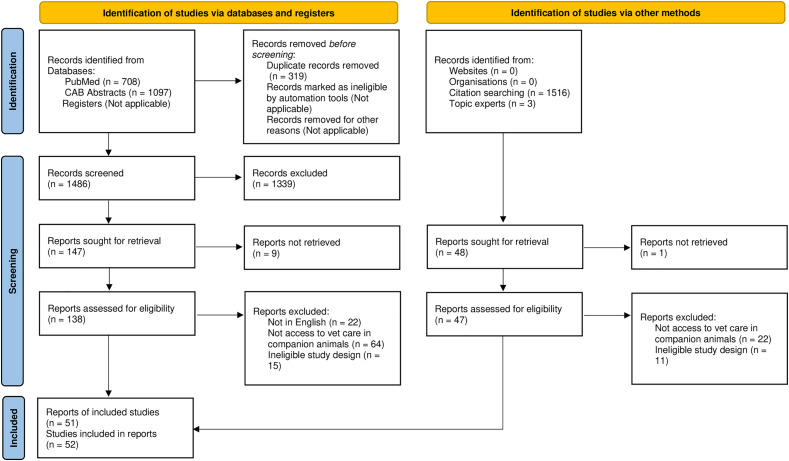
PRISMA flow diagram [21] of the number of records identified, screened, and included in a scoping review of risk factors for, metrics of, and consequences of access to veterinary care for companion animals.

The publisher apologizes for the error.
