# Supplementary material for: Risk factors for, metrics of, and consequences of access to veterinary care for companion animals: A scoping review
Source: PLoS One. 2025 May 30;20(5):e0325455. doi: 10.1371/journal.pone.0325455 (PMC12124545; doi:10.1371/journal.pone.0325455)
Supplement: S1 Table — (DOCX) [file pone.0325455.s001.docx]

**S1 Table. Search strings used in a CAB Abstracts search conducted on 13 July 2023 for a scoping review of risk factors for, metrics of, and consequences of access to veterinary care in companion animals.**

| String | Search | Number of hits |
| --- | --- | --- |
| 1 | TS=(veterinary OR cat OR dog OR pet Or "companion animal" OR canine OR feline OR mule OR donkey ) | 583,200 |
| 2 | TI=("veterinary deserts" OR "access to veterinary care" OR "access to care" OR "low income" OR underserved OR Unserved OR socioeconomic) | 11,997 |
| 3 | AB=("veterinary deserts" OR "access to veterinary care" OR "access to care" OR "low income" OR underserved OR Unserved OR socioeconomic ) | 76,254 |
| 4 | #2 OR #3 | 79,030 |
| 5 | #1 AND #4 | 1,097 |
